# Clinical, radiological and laboratory findings in 185 children with tuberculous meningitis at a single centre and relationship with the stage of the disease

**DOI:** 10.1186/s13052-015-0186-7

**Published:** 2015-10-15

**Authors:** Ali Güneş, Ünal Uluca, Fesih Aktar, Çapan Konca, Velat Şen, Aydın Ece, Salih Hoşoğlu, Mehmet Ali Taş, Fuat Gürkan

**Affiliations:** Medical School Department of Pediatrics, Dicle University, Diyarbakir, Turkey; Medical School Department of Infectious Diseases, Dicle University, Diyarbakir, Turkey; Medical School Department of Pediatrics, Adiyaman University, Adiyaman, Turkey; Medical School Department of Pediatric Pulmonology, Dicle University, Diyarbakir, Turkey

**Keywords:** Children, Tuberculous meningitis, Stage, Diagnosis, Findings

## Abstract

**Background:**

A delay in the diagnosis and treatment of tuberculosis meningitis (TBM) may lead to increased mortality and morbidity. The aim of this study was to describe the clinical, radiological and laboratory findings of TBM on a cohort of 185 pediatric patients at a single centre over a 10 year period and to investigate relationship between the stage of the disease.

**Methods:**

The hospital records of 185 TBM children that presented to the Pediatric Clinics of Dicle University Hospital were retrospectively evaluated. The age, gender, family history of tuberculosis, result of Mantoux skin test, status of BCG vaccination, stage of TBM at hospitalization, and clinical, laboratory and radiological features were recorded. Clinical staging of TBM was defined as follows: Stage I, no focal neurological findings and Glasgow Coma Scale (GCS) score 15; Stage II, GCS 15 presenting with focal neurological deficit or all the patients with GCS 10–14; Stage III, all the patients with GCS < 10. Relationships between results and stages of TBM were investigated.

**Results:**

The mean age of the patients was 53.5 ± 44.9 months (4 months–18 years). 121 (65.4 %) of the patients were male and 64 (34.6 %) female. Family history of tuberculosis was defined in 62 (33.5 %) patients. Forty five (24.3 %) children had BCG vaccination scar. Mantoux skin test was interpreted as positive in 35 (18.9 %) patients. Sixty-eight (36.8 %) children were at stage I TBM, 57 (30.8 %) at stage II and 60 (32.4 %) were at stage III on admission. Mean duration of hospitalization was 23.9 ± 14.1 days. Totally, 90 patients (48.6 %) had abnormal chest X-ray findings (parenchymal infiltration in 46 (24.9 %), mediastinal lymphadenopathy in 36 (19.5 %), miliary opacities in 25 (13.5 %), pleural effusion in 2 (1.1 %), and atelectasis in 2 (1.1 %) patients). One hundred sixty seven (90.3 %) patients had hydrocephalus in cranial computerized tomography. There were 24 (13.0 %) patients with positive culture for Mycobacterium tuberculosis and 3 (1.6 %) patients with positive acid-fast bacilli in cerebrospinal fluid. Overall mortality rate was 24 (13.0 %). Among the findings; patients at Stage III had less frequent positive chest X-ray abnormality, miliary opacities and BCG vaccination scar when compared with patients at Stage I and II (*p* = 0,005; *p* = 0,007, *p* = 0.020, respectively).

**Conclusions:**

Children with TBM and positive chest X-ray findings at hospital admission were more frequently diagnosed at Stage I, and BCG vaccination might be protective from the Stage III of the disease.

## Introduction

According to the World Health Organization, in 2013, approximately 9 million cases of tuberculosis (TB) were detected with 1.5 million of them resulting in death. Approximately 10 % of these patients were children and 15 % of these patients presented with extra pulmonary tuberculosis [1]. Central nervous system tuberculosis is an extra-pulmonary tuberculosis with higher mortality and morbidity. Tuberculosis meningitis (TBM) is the most frequently observed form of central nervous system TB [2]. Although, TBM can be treated completely by early diagnosis and treatment, three quarters of the patients die if treatment is delayed [3]. The definitive diagnosis of TBM is established by the detection and/or the culture of *Mycobacterium tuberculosis* in the cerebrospinal fluid (CSF) [4]. The necessity of long time for *Mycobacterium* growth on specific culture medium and the low possibility of direct *Mycobacterium* identification with CSF smear staining may lead to the delay in diagnosis. In order to prevent the delays in identification, the utilization of auxiliary identification techniques such as nucleic acid amplification (NAA), Polymerase Chain Reaction (PCR), antibody detection, and antigen detection have been used [4, 5]. Diagnostic algorithms have been tried to implement by using some clinical, laboratory and imaging data, especially in developing countries, where the auxiliary diagnosis techniques are expensive and mostly inaccessible [4]. Tuberculous meningitis mostly develops within 2–6 months following primary pulmonary infections during childhood [6]. Therefore, the determination of pulmonary TB findings may be useful for the early diagnosis of TBM [4]. Interpretation of epidemiological, clinical, laboratory and radiological findings among a large series of patients with TBM may be beneficial for the early diagnosis of the disease. This presented study aimed to describe the clinical, laboratory and radiological features of 185 pediatric patients with TBM hospitalized at our clinics over a 10 year period and to investigate the relationship of these features between the stages of the disease.

## Methods

The hospital records of children admitted to the Department of Pediatrics of Dicle University Hospital in Diyarbakir Turkey between 1998 and 2008 and diagnosed as TBM have been evaluated retrospectively. Twelve patients were excluded due to insufficient data in their files, therefore 185 children were assessed. Age, gender family history of TB, clinical, laboratory, and radiological findings, Mantoux skin test results, Bacillus Calmette-Guerin (BCG) vaccination status and the history of close contact with TB disease were recorded. Mantoux skin test was considered positive in patients with endurance diameter > 15 mm in BCG scar positive patients and > 10 mm in BCG scar negative patients [7]. The chest X-rays of the patients were evaluated by a pediatrician. Patients with mediastinal lymphadenopathy, parenchymal infiltration, and miliary opacities were evaluated favor of TB. All patients were performed cranial computed tomography (CT) to diagnose possible ventricular enlargement. Diagnosis of TBM was done according to ‘Uniform TBM resource case definition’ criteria (Table 1) [4]. The patients who did not fulfill the criteria, age of < 1 month and > 18 years have been excluded from the study. The determination of the clinic stages of TBM was done according to the criteria of Medical Research Council [8] as follows: Stage I, patients with no focal neurological findings and Glasgow Coma Scale (GCS) score 15;Stage II, patients with GCS 15 that presenting with focal neurological deficit or all the patients with GCS between 10 and 14, regardless of the presence of focal neurological deficit; Stage III, all the patients with GCS < 10. Cerebrospinal fluid, fasting gastric juice and sputum were obtained for acid-fast bacilli (AFB) stain and culture. Lowenstein-Jensen medium was used for culture. The study protocol was approved by the Non-interventional Ethical Committee of Dicle University Medical School.

**Table 1:** Diagnostic criteria for classification of definite, probable, possible, and not tuberculous meningitis^4^

	Diagnostic score
Clinical criteria (Maximum category score = 6)	
Symptom duration > 5 days	4
Systemic symptoms suggestive of TB (one or more of the following): weight loss (or poor weight gain in children), night sweats, or persistent cough > 2 weeks	2
History of recent (within past year) close contact with an individual with pulmonary TB or a positive TST or IGRA (only in children <10 years of age)	2
Focal neurological deficit (excluding cranial nerve palsies)	1
Cranial nerve palsy	1
Altered consciousness	1
CSF criteria (Maximum category score = 4)	
Clear appearance	1
Cells: 10–500 per μl	1
Lymphocytic predominance (>50 %)	1
Protein concentration > 1 g/L	1
CSF to plasma glucose ratio < 50 % or an absolute CSF glucose concentration < 2.2 mmol/L	1
Cerebral imaging criteria (Maximum category score = 6)	
Hydrocephalus	1
Basal meningeal enhancement	2
Tuberculoma	2
Infarct	1
Pre-contrast basal hyperdensity	2
Evidence of TB elsewhere	(Maximum category score = 4)
Chest radiograph suggestive of active TB: signs of TB = 2; miliary TB = 4	2/4
CT/ MRI/ ultrasound evidence for TB outside the CNS	2
AFB identified or *M.tuberculosis* cultured from another source—ie, sputum, lymph node, gastric washing, urine, blood culture	4
Positive commercial *M.tuberculosis TB* NAAT from extra-neural specimen	4

Exclusion of alternative diagnoses: An alternative diagnosis must be confirmed microbiologically (by stain, culture, or NAAT when appropriate), serologically (eg, syphilis), or histopathologically (eg, lymphoma). The list of alternative diagnoses that should be considered, dependent upon age, immune status, and geographical region, include: pyogenic bacterial meningitis, cryptococcal meningitis, syphilitic meningitis, viral meningo-encephalitis, cerebral malaria, parasitic or eosinophilic meningitis (*Angiostrongylus cantonesis*, *Gnathostoma spinigerum*, toxocariasis, cysticercosis), cerebral toxoplasmosis and bacterial brain abscess (space-occupying lesion on cerebral imaging)and malignancy (eg, lymphoma) *TB* tuberculosis, *TST* tuberculin skin test, *IGRA* interferon-gamma release assay, *NAAT* nucleic acid amplification test, *AFB* acid-fast bacilli

### Statistical analysis

Data was presented as mean plus/minus standard deviation or number and percentages. Comparison of subgroups were done by Chi-square test. *P* value less than 0.05 was accepted as statistically significant.

## Results

Among the 185 patients enrolled to the study, 121 (65.4 %) were boys and 64 (34.6 %) were girls. The mean age of the patients was 53.5 ± 44.9 months (range: 4 months – 18 years). A history of close contact with an adult TB was reported in 62 patients (33.5 %). Thirty-five patients (18.9 %) presented with positive Mantoux skin test result, and 45 patients (24.3 %) presented with positive BCG vaccination status (38.5 % in Stage I, 46.2 % in Stage II and 15.4 % in Stage III; *P* = 0.020). The most frequent preadmission symptoms were fever in 144 patients (78.7 %) and vomiting in 124 patients (70.1 %). Sixty-eight (36.8 %) children were at stage I TBM, 57 (30.8 %) at stage II and 60 (32.4 %) were at stage III on admission.

In cranial CT, hydrocephaly was found in 167 patients (90.3 %). Twelve (6.6 %) patients presented with tuberculoma. The chest X-ray imaging revealed abnormal imaging findings in 90 (48.6 %) patients (parenchymal infiltration in 46 (24.9 %), lymphadenopathy in 36 (19.5 %), miliary opacities in 25 (13.5 %), pleural effusion in 2 (1.1 %), and atelectasis in 2 (1.1 %) patients). Pulmonary hilar lymphadenopathy was observed in 21(11.4 %), paratracheal lymphadenopathy in 10 (5.4 %), subcarinal lymphadenopathy in 3(1.6 %) and paraaortic lymphadenopathy in two patients (1.1 %). Among the patients with miliary opacities, 64.0 % were at stage I (*P* = 0.007).

There were 24 (13.0 %) patients with positive culture for MTb, 3 (1.6 %) patients with positive AFB in cerebrospinal fluid and 1 (0.5 %) patient with positive AFB in fasting gastric juice. Nine patients (4.8 %) had positive polymerase chain reaction (PCR) for MTb in CSF.

Above clinical, laboratory and radiological findings were compared between patients at different stages of the disease (Tables 2, 3, 4). Patients at Stage III had less frequent positive chest X-ray abnormality, miliary opacities and BCG vaccination scar when compared with the patients at Stage I and II (*P* = 0,005; *P* = 0,007, *P* = 0.020 respectively).

**Table 2:** Relationship between clinical stage of tuberculosis meningitis and laboratory findings

	Stage 1	Stage 2	Stage 3	*p*
	Mean ± standard deviation	Mean ± standard deviation	Mean ± standard deviation	
Blood leukocytes (mm^3^)	12.2 ± 5.6	14.3 ± 9.7	13.4 ± 7.6	0.770
ESR (mm/hour)	43.5 ± 32.6	36.5 ± 22.5	41.0 ± 25.6	0.863
Serum Sodium level (mEq/L)	132.7 ± 7.4	128.9 ± 8.5	131.4 ± 7.6	0.198
CSF protein (mg/dl)	96.9 ± 105.7	93.9 ± 105.6	122.99 ± 122.9	0.075
CSF/serum glucose (mg/dl)	0.47 ± 0.19	0.27 ± 0.14	0.27 ± 0.13	0.054
CSF leukocytes count (/mm^3^)	239.5 ± 232.1	253.3 ± 263.9	212.8 ± 223.8	0.330
Hemoglobine (gr/dl)	11.1 ± 2.3	10.9 ± 1.8	10.8 ± 1.9	0.709

*ESR* erythrocyte sedimentation rate, *CSF* cerebrospinal fluid

**Table 3:** Relationship between clinical stage of tuberculosis meningitis and chest radiography findings

Chest radiography findings	Stage I	Stage II	Stage III	*p*
	(*n* = 68) *n* (%)	(*n* = 57) *n* (%)	(*n* = 60) *n* (%)	
Abnormal chest radiography	40 (44.4)	31 (34.4)	19 (21.1)	0.005
Parenchymal involvement	18 (39.1)	18 (39.1)	10 (21.7)	NS
Miliary opacities	16 (64.0)	6 (24.0)	3 (12.0)	0.007
Mediastinal lymphadenopathy	14 (38.9)	11 (30.6)	11 (30.6)	NS
Atelectasis	1 (50.0)	0 (0.0)	1 (50.0)	NS
Pleural effusion	0 (0.0)	0 (0.0)	2 (100.0)	NS

*NS* not significant

**Table 4:** Relationship between clinical stage of tuberculosis meningitis and clinical findings

Clinical findings	Stage I	Stage II	Stage III	*p*
	(*n* = 68) *n* (%)	(*n* = 57) *n* (%)	(*n* = 60) *n* (%)	
Neck rigidity	40 (21.6)	36 (19.6)	30 (16.2)	0.168
Vomiting	36 (19.5)	42 (22.7)	43 (23.2)	0.922
Fever	41 (22.2)	50 (27.0)	51 (27.6)	0.720
Headache	25 (13.5)	20 (10.8)	17 (9.2)	0.062
Consciousness	1 (0.5)	62 (33.5)	67 (36.2)	<0.001
Convulsion	16 (8.7)	25 (13.5)	41 (22.2)	0.001

Anti-tuberculous therapy including isoniazid and rifampin were used for a total of 12 months together with an initial treatment of streptomycin, pyrazinamide or ethambutol for the first 2 months of treatment. Steroid was given to all patient in the first month tapered by the following days of treatment and other measures including mannitol and anticonvulsants were administered when necessary. Mean duration of hospitalization was 23.9 ± 14.1 days, and twenty-four patients (13.0 %) died during the follow-up.

## Discussion

In TBM patients, early diagnosis and adequate treatment are the most important factors that affect mortality and morbidity rates. The definitive diagnosis of TBM is made by the growth of the bacteria in Lowenstein-Jansen solid medium culture and/or the observation of ARB in CSF with the microscopic examination of CSF smear identified by Erlich-Ziehl-Nielsen stain. The rates of direct observation of bacteria and/or the bacterial growth in culture present important differences in the literature [9]. The positive culture result depends on the repeating puncture number, the amount of CSF taken, and the capability of laboratory conditions [4, 10]. In previous studies, the ratio of positive culture for *M. tuberculosis* from CSF varies between 10-71 % [10–12]. In our present study, the ratio of positive CSF culture for *M. tuberculosis* was 13 % in TBM patients. The sum of positive culture result, ARB direct visualization in stained CSF smear, and positive PCR ratio was 20.0 % in our patients. The low rate of positive CSF culture and direct visualization of ARB may be related to low number of repeated lumbar puncture and small amount of CSF obtained. Because, the number of repeating lumbar puncture and the collection of big amount of CSF is not always possible in children. Therefore, the utilization of different diagnostic modalities such as nucleic acid amplification (NAA) method, PCR, antibody detection, antigen detection, adenosine deaminase (ADA), and tuberculostearic acid measurement are recommended [13]. As the access to these auxiliary methods may be difficult, their utilization is relatively limited in developing countries where TBM is quite prevalent. As a result, these limitations may lead to the diagnosis of the TBM disease at later stages. In order to prevent diagnostic delays, attempts have been made to create diagnostic rules based on clinical, laboratory and radiological observations [4]. One hundred eight (% 58.4) of our patients were under 5 years of age. Nonspecific but consistent clinical, laboratory and radiological findings should be more carefully evaluated below 5 years this age. One hundred twenty one (65.4 %) of the patients were male and there was a family history of tuberculosis in 62 patients. Mantoux skin test is a significant diagnostic method for childhood tuberculosis and it was found positive in only 35 (18.9 %) of our patients. The most frequent preadmission symptoms were fever (78.7 %) and vomiting (70.1 %). Although HIV serology tests were absent in our patients, HIV infection was not suggested as a predisposan factor in our patients, because of the very low prevalence of HIV in children and even in adults in Turkey, and especially in our region.

In most studies, three quarters of the patients were at stage II or III and those results have supported delaying in diagnosis [5, 14–16]. In a previously reported review of 214 children with TBM at our unit in the past decade during 1998 and 2006, there were 22 (10 %) patients in Stage I, 120 (56 %) patients in Stage II and 72 (34 %) patients in Stage III [17]; while in our present study there were 68 (37 %) patients in Stage I, 57 (31 %) patients in Stage II and 60 (32 %) patients in Stage III of the disease. In comparison of these two study periods (1988–1996 and 1998–2008), according to new policies of Ministry of Health, people could reach more advanced health facilities, there was more awareness of infectious diseases and vaccination policies were more strict. Because of the above reasons, in comparison with the previous study, there were more patients diagnosed at Stage I (37 % vs 10 %). There were also more patients with positive BCG vaccination in our series than the previos one (24 % vs 12 %). In our patients there was statistically significant difference between the stages of the disease regarding BCG vaccination status (38.5 % positive in Stage I, 46.2 % in Stage II and 15.4 % in Stage III; *P* = 0.020). The protection rate of BCG vaccination in tuberculosis disease is not well known (0 % to 80 %), but its role against severe forms of tuberculosis including TBM and miliary tuberculosis has generally been accepted [18]. The relatively low incidence of patients with BCG scar in Stage III patients in our study could also suggest that BCG vaccination might be protective from the more severe Stage III of TBM.

Chest radiography findings (especially miliary tuberculosis) are among the diagnostic criteria used for diagnosis of TBM in clinical studies [10]. In previous studies, ratios and types of abnormal chest X-ray findings in TBM patients showed considerable differences. While the chest X-ray findings favoring pulmonary TB were found in 66 % and 60 % of TBM patients in different studies [5, 19], in a recent prospective study the conclusion was that the majority of the children with TBM, including the very young, did not have signs suggestive of TB on chest x-ray [20]. Approximately half of our patients (52 %) did not have chest X-ray abcormalities. There were parenchymal infiltration in 24.9 %, lymphadenopathy in 19.5 %, miliary opacities in 13.5 %, pleural effusion in 1.1 %, and atelectasis in 1.1 % of our patients. Lymphadenopathy might have been overlooked being superposed on pulmonary parenchymal infiltration. Thorax computed tomography has been reported to be more sensitive to show lymphadenopathy [21].

Definitely, chest X-ray alone is insufficient in diagnosing early TBM. However especially milliary opacities is an established risk factor for TBM. A previous study showed TBM in approximately one third of the patients with miliary tuberculosis [22]. Another post-mortem study reported that miliary tuberculosis was present in most of the TBM patients [23]. Among our patients with miliary opacities, 64.0 % were at stage I (*P* = 0.007). Moreover, significantly higher ratio of abnormal chest radiography findings was found in stage I TBM patients while comparing with stage II and III patients in the present study. More focus should be needed for diagnosis of tuberculosis in patients with neurological symptoms at Stage III TBM, since these patients had less frequent signs of pulmonary tuberculosis on radiography.

A standart anti-tuberculous treatment including isoniazid and rifampin for 12 months, and an additional initial treatment of streptomycin, pyrazinamide or ethambutol for the first 2 months were given to all patients together with glucocorticoid therapy for the first month tapered on the following days. Hydrocephaly was found in 87 % patients. Mannitol, anticonvulsant therapy and surgical management were applied when necessary. Overall mortality rate was 13 % in our series, less than the previously reported series in our department (23 %), although there was no change on the treatment strategy.

## Conclusion

Children with TBM and positive chest X-ray findings at hospital admission were more frequently diagnosed at Stage I, and patients at Stage I had more frequent BCG vaccination scar in our study. Diagnosis of patients with TBM at earlier stages is crucially important to decrease mortality rates besides appropriate treatment strategies. More focus of the doctors on diagnosis of tuberculosis when patients presenting with early neurological symptoms of meningitis without abnormal chest x-ray findings are encountered and increasing BCG vaccination availability of the patients might increase number of patients diagnosed at earlier stages of TBM.
